# “Medical Student Syndrome”—A Myth or a Real Disease Entity? Cross-Sectional Study of Medical Students of the Medical University of Silesia in Katowice, Poland

**DOI:** 10.3390/ijerph18189884

**Published:** 2021-09-19

**Authors:** Katarzyna Szczurek, Natalia Furgał, Dawid Szczepanek, Rashid Zaman, Krzysztof Krysta, Marek Krzystanek

**Affiliations:** 1 Department of Psychiatric Rehabilitation, Department of Psychiatry and Psychotherapy, Faculty of Medical Sciences in Katowice, Students’ Scientific Association, Medical University of Silesia, 40-635 Katowice, Poland; katarzyna.szczurek@interia.eu (K.S.); natalia.furgal@gmail.com (N.F.); szczepanekdawid@interia.pl (D.S.); 2 Department of Psychiatry, University of Cambridge, Cambridge CB2 0SZ, UK; rz218@cam.ac.uk; 3 Department of Psychiatric Rehabilitation, Department of Psychiatry and Psychotherapy, Faculty of Medical Sciences in Katowice, Medical University of Silesia, 40-635 Katowice, Poland; krzystanekmarek@gmail.com

**Keywords:** hypochondria symptoms, nosophobia symptoms, medical students

## Abstract

The description of Medical Student Syndrome is based on the assumption that inexperienced medical students are prone to develop a pathological fear of medical conditions they are taught about. The aim of this study is to examine the sample of students (medical and non-medical) in order to assess and compare their level of hypochondriacal attitudes and health-related anxiety. We also examined other factors which might have had an influence on hypochondria and nosophobia attitudes among students. **Methods:** The study was conducted in two groups of students: 313 medical students at the Medical University of Silesia and 293 students at non-medical universities in Katowice, Poland. The study used the medical student syndrome self-explanatory questionnaire constructed for the study, taking into account the specificity of the group and the research problem. The research questionnaire was completed in an online survey by 606 students. **Results:** The results of the study showed that medical students obtained the same scores on a nosophobic scale as the non-medical students (*p* = 0.5). The analysis of hypochondriacal behavior showed significantly higher results in the non-medical student group (*p* = 0.02). In the entire study group, females and participants with mental disorders obtained higher scores in relation to nosophobia. Symptoms of depression and anxiety were more common in the group of medical students. **Conclusions:** Medical studies are not a risk factor for the occurrence of health anxiety and hypochondrial attitudes. Such factors are female gender and having a mental illness.

## 1. Introduction

For a number of decades, a widely held belief about the Medical Students Syndrome (also known as medical students’ disease), affecting young people studying medicine, has been circulating in medical universities [1,2,3,4,5,6]. It has been theorized that learning about life-threatening ailments in the course of medical training is associated with a risk of developing anxiety or illness anxiety disorder in which there is an uncontrollable and persistent fear of having a serious medical condition, described as nosophobia [2].

Exposing medical students to a large amount of clinical knowledge about different diseases may result in a search for symptoms affecting them, worrying about having a serious illness or an exaggeration of minor symptoms, which may lead to a self-diagnosis of a specific somatic disease; a condition called hypochondria [3]. On the other hand, having not enough knowledge and experience may result in incorrect diagnoses. The reasons for the emergence of such a phenomenon may include exposure to the symptoms of patients, with whom students come into contact, exposure to a competitive and demanding environment that causes stress, and students’ personal emotional reactions [1,7]. One of the elements, which may determine individual response to stress amongst some, and which plays a role in causing medically unexplained symptoms and fear of diseases are alexithymic personality traits [8,9]. Because of the fact that the classification of psychosomatic disorders is not standardized and can cause confusion, for the purpose of our study we decided to clarify the terms used in our research [10]. 

We applied the following definitions. “Nosophobia” is the extreme or irrational fear of developing or having a specific disease “ [11]. The term “hypochondria” refers to Hypochondriasis—“a psychiatric disorder involving a preoccupation with fears of having, or the idea that one has, a serious disease based on the misinterpretation of bodily symptoms despite appropriate medical evaluation and reassurance” [11,12]. Young medical students are exposed to severe stress imposed by the environment [2,13,14,15]. They are also more likely to suffer from depression, anxiety and other mental disorders than their peers [1,16,17]. Is medical students’ disease another mental illness that this population is more exposed to in comparison with other groups? The aim of our study was to assess the level of hypochondria and nosophobia present in the groups of medical students and non-medical students and to evaluate the differences if any. 

This issue is very important since this phenomenon was described as early as the 1960s. It was postulated that it affects 70–80% of students, however, these studies from 1964 and 1966 [2,3] were conducted on relatively small numbers (77 and 33 respondents) and were not standardized. On the contrary, there are reports of relatively newer research (from 1998 and 2014), conducted on groups of students from Great Britain [4,6], which did not show a significant difference in the level of nosophobia and between medical and non-medical students. Conclusions contradictory to those mentioned above have been published by Ellingsen and Wilhelmsen (2002), who examined a population of 80 medical students and compared them with 100 law students, and observed a lower level of fear of illness in the medical student group [5]. Due to contradictory literature and lack of data on the situation in Poland, we decided to study the student population of universities in the Silesian Agglomeration, comparing the results obtained from medical and non-medical students. Before we designed the study, we organized a focus group interview with the participation of students from different universities in our region, during which we asked them how important for them is the issue of having a fear of a serious disease. The unpublished results of this interview led to the conclusion that this topic is very important for both medical and non-medical students. The participants of the focus group agreed that there is a need for an increased psychological and psychiatric support for students in our region, and that the results of the study we planned, could be a tip for the regional and academic authorities helping to decide, how to organize the health policy and health education for young people studying in the universities of our Agglomeration. 

An additional factor that we considered was the COVID-19 pandemic, which increased the general public awareness of the possibility of contracting a disease, and also contributed to the increase in the frequency of anxiety and depression [18,19,20,21,22,23]. For many, it led to isolation from family and friends and limited involvement in social activities. The pandemic forced the students to stay in their place of residence, the majority of teaching was delivered remotely, which could also exacerbate anxiety, deficits in concentration and disruptions of sleeping patterns [24,25]. Isolation has an impact not only on people with mental disorders [26]. One of the groups particularly vulnerable to such a situation are students [27,28,29] and medical staff [30]. The conclusions of the pre-COVID-19 pandemic research emphasized that quarantine may be a catalyst for mental health problems in individuals, who did not have problems in this area before [31]. With this in mind, we also investigated whether the pandemic situation could also have contributed to the incidence of nosophobia and hypochondria symptoms.

## 2. Materials and Methods

The study was conducted based on the medical student disease questionnaire we designed and distributed amongst respondents, who were users of Facebook groups. These groups were private and opened only for a specified group of students. The participants were informed about the purpose of the study and undertook to participate in it voluntarily. The data were collected from November 2020 to January 2021. Since there is no existing questionnaire dedicated to medical student disease, a separate self-explanatory questionnaire was developed, based on diagnostic criteria of Illness Anxiety Disorder (300.7; DSM-5) (F45.21; ICD-10), hypochondria (F45.2; ICD-10) and phobias (F40; ICD-10) [32], containing questions about the symptoms of hypochondria and nosophobia. The questionnaire consisted of two parts. The complete questionnaire is available in Supplementary materials as a Questionnaire S1. Participants suspected of suffering from medical student disease were expected to perform excessive health-related behaviors and exhibit maladaptive avoidance. The last criterion was the absence of other mental disorders, which could better explain the illness-related preoccupation. 

In order to better illustrate the problem and to refer to the results of previously conducted research studies discussed in the Introduction section, the questions were assigned to the symptoms of hypochondria and nosophobia. Due to the form of delivering the questionnaires, it was necessary to control the correctness of filling them in. For this purpose, inverted questions were used. The questionnaire was completed by a random sample of 606 respondents studying in the Silesian Region. 

The medical student group consisted of 313 students of all years of the medical faculty of the Medical University of Silesia in Katowice. The non-medical student group consisted of 293 students of all years of various fields of academic education not related to medicine (e.g., Administration, Law, IT, Philology, Pedagogy, Construction, Management and almost 50 other fields) studying at the Silesian University of Technology, University of Silesia in Katowice or University of Economics in Katowice. Out of the group of non-medical students, the responses obtained from psychology students were excluded, as it was concluded that in some respects, their knowledge may coincide with the knowledge of medical students. The study was granted exemption from requiring ethics approval after consultation with the Bioethics Committee of the Medical University of Silesia.

### 2.1. Cross-Section of the Group 

The sample consisted mainly of women, representing 73.4% (n = 445) of the entire cohort. Males accounted for 26.6% (n = 161). The median age was 22 years for the medical student’s group (range 18–30 years) and 21 years for the non-medical student group (range 18–42 years). In the medical student’s group, the largest part of the respondents were fourth-year students—27.5% (n = 86) and first-year students-26.8% (n = 84). Among the students of non-medical faculties, there were 42.3% (n = 124) of first-year students (Table 1). 

### 2.2. Statistical Methods

The presented analyzes were performed in the Statistica 13.3 program. A significance level of Alpha < 0.05 was adopted for the total statistical summary. In the search for answers to the research questions, the analysis of differences with Student’s *t*-tests and the Spearman’s rho rank correlation coefficient were used. In the additional analysis, the inequality of the respondents in the groups requires the use of a non-parametric Mann–Whitney U test. The whole evaluation was supplemented with the analysis of the response frequency and the results of the chi-square test of independence.

Table 2 provides the statistical description of the variables used. On its basis, we can state that the tested variables are not compatible with the normal distribution and that the results of hypochondria and nosophobia symptoms are right-skewed. This results in a slight advantage of the number of lower scores over higher scores, and the positive kurtosis of variables during the pandemic suggests a higher level of concentration of scores around the mean. The pandemic fear variables also show slight right-skewness, but a greater dispersion of results.

## 3. Results

### 3.1. Analysis of the Level of Hypochondria and Nosophobia Symptoms 

The first analyzed problem is the difference in the level of hypochondria and nosophobia symptoms between students of medical and non-medical faculties—this is illustrated in Table 3. Despite a higher score on the scale of potential nosophobia in the group of medical students, who scored an average of 12.52 points, this difference in comparison to non-medical students with an average of 12.28 points, is statistically not significant (*p* = 0.5). The presented analysis suggests that a group of medical students and a group of non-medical students achieve similar results of nosophobia. The analysis of the responses to hypochondriacal behaviors showed that students of non-medical faculties achieved an average of 13.38 points, compared to medical students M = 12.55, which is a significantly higher result (*p* = 0.02). However, the strength of the indicated effect is weak.

### 3.2. The Correlation between the Level of Nosophobia and Hypochondria Symptoms and the Year of Study

Another aspect of the study of the differences in the intensity of nosophobia and hypochondria symptoms was the evaluation of the correlation with the Spearman’s rho coefficient of the level of hypochondria symptoms and nosophobia in relation to the year of study. The analysis was performed both in the entire study group and in subgroups of students of medical and non-medical faculties. The results are presented in Table 4. The rank correlation coefficient was selected due to the scale of measurement of the variables. 

With regard to the entire study group and the group of students of non-medical faculties, there is no significant relationship between the level of nosophobia and hypochondria and the year of study (*p* = 0.07 in the group of non-medical faculties and *p* = 0.478 in relation to the entire cohort). In the group of medical students, the correlation between the year of studies and the level of hypochondria symptoms also turned out to be insignificant (*p* = 0.238). Only the year of studies in the group of medical students turned out to be significantly correlated with the level of nosophobia (*p* = 0.026). The higher the stage of academic education of a medical student, the higher the results of nosophobia obtained. However, it must be remembered that this is a weak correlation.

### 3.3. Gender Influence on the Studied Variables

An interesting aspect of the work is to investigate the differences in the level of hypochondria and nosophobia symptoms between women and men in both groups. For this purpose, the Mann-Whitney U test was used. This analysis in the entire study group and in the subgroups is presented in Table 5. For the groups, which are not equal in number, results can be presented that indicate that both in the entire group and in individual groups of students of medical and non-medical faculties, women achieve higher results in the level of hypochondria and nosophobia. Those in non-medical fields, women obtained significantly higher results (*p* = 0.009 for hypochondria and *p* = 0.005 for nosophobia). In the case of the respondents studying medicine, a significantly higher result for women was obtained when trying to assess nosophobia (*p* = 0.03). On the other hand, the results of hypochondria symptoms presented by medical students in both sexes are comparable (*p* = 0.996), *r_g_* = 0.00. In the entire study group, the female sex received significantly higher results in relation to the fear of developing the disease (*p* = 0.001).

### 3.4. Linking Psychiatric Treatment with the Severity of Nosophobia and Hypochondria Symptoms

The respondents were asked about their mental health, visiting a psychiatrist or readiness to undertake psychiatric treatment. Results showed that a third of the students (33%) out of three non-medical universities in Katowice, declared that they suffered from a mental illness. The most common disorder in this group is depression, which accounted for 40% of all disorders declared by students of non-medical faculties. An equally frequent disorder in this group (38%) is anxiety and anxiety-depressive disorders. Of respondents in this group, 20% benefited from psychiatric treatment related to the above mental disorders. 

In a group of 313 medical students, 85 individuals (27%) declared having a mental disorder. In this study group, anxiety and anxiety-depressive disorders predominate, affecting 54% of individuals with mental disorders. Depression is in second place (24.7%). In this group, 14% of respondents took advantage of help from psychiatrists. The results described above are presented in Table 6.

Due to the high frequency of mental disorders in the studied population, it was decided to investigate the correlation of the studied variables with the psychiatric treatment undertaken for the diseases declared by the respondents. The analysis was carried out with the Mann–Whitney U test between the examined psychiatric patients and non-psychiatric ones, in the whole group and divided into the research and control group. Table 7 presents the results of the analysis. In the whole study group (*p* < 0.001), as well as in the medical student group (*p* < 0.001) and the non-medical student group (*p* = 0.023) individuals who took advantage of psychiatric treatment achieved significantly higher results of nosophobia than students who did not visit psychiatrists. In the case of hypochondria symptoms, the results also indicate its higher level in the case of individuals using psychiatric help (*p* <0.001 for the entire cohort, *p* = 0.003 for the medical students and *p* = 0.011 for non-medical students). For the variables of nosophobia and psychiatric treatment in the group of medical students, the effect size is moderate (rg = 0.38). 

**Figure 1:**
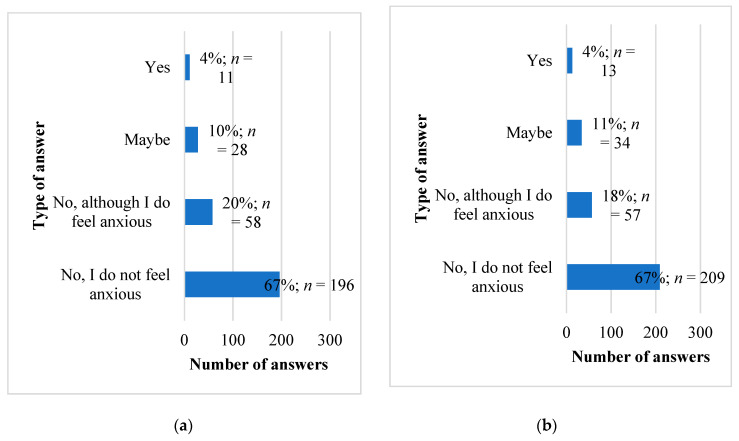
Number of responses of the surveyed students of non-medical (**a**) and medical (**b**) faculties to the question about the planned visit to a psychiatrist in connection with the anxiety experienced. (**a**) Number of observations *n* = 293. (**b**) Number of observations *n* = 313.

The respondents were also asked about a planned visit to a psychiatrist due to the fear of falling ill. Both groups responded similarly, as shown in Figure 1. From the respondents, 20% of non-medical students and 18% of medical students indicated that they will not contact a psychiatrist despite feeling anxious. The most common reason for not wanting to visit the psychiatrist was the feeling that they would not be able to cope with the anxiety on their own. On the other hand, as many as seven respondents from the medical student group were afraid of stigmatization related to the treatment undertaken, while in the non-medical group there were only four such responses.

In view of the prevailing COVID-19 pandemic, respondents were asked about the declaration of the undertaking of psychiatric treatment regarding their fears they suffered from during the pandemic as well as before it started. The responses of both groups (Table 8) showed that the number of students willing to start psychiatric treatment of perceived anxiety decreased. The number of declarations to contact a psychiatrist during a pandemic is lower than before the COVID-19 pandemic.

The difference between the number of non-medical students taking advantage of a psychiatrist’s help due to their fear of developing a disease before the pandemic (6.5%) compared to the period during the COVID-19 pandemic (2.7%) decreased significantly (*p* = 0.034). In the group of medical students, the difference was insignificant (*p* = 0.3).

### 3.5. Observation of Disease Symptoms during Clinical Classes 

**Figure 2:**
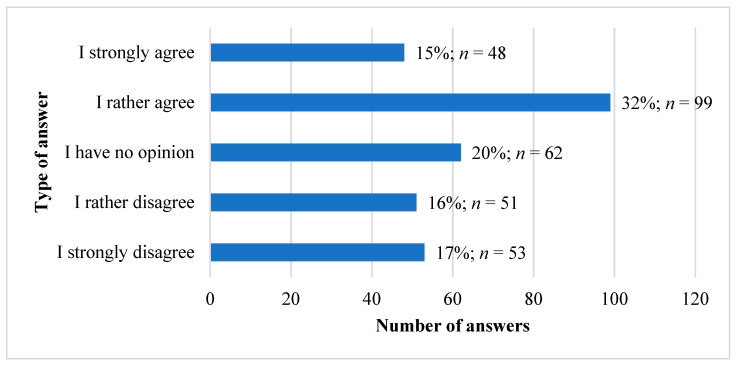
Number of responses of the surveyed medical students to the question about paying more attention to symptoms of diseases after completing clinical classes on a given disease entity. *n* = 313.

In addition to the above analysis, we attempted to explore if there was any change in the frequency of attention paid to the symptoms after learning about a particular clinical disease. Figure 2. presents the responses of the surveyed medical students to the question about paying more attention to potential symptoms of diseases after becoming familiar with them after the clinical classes. Forty-seven percent of the surveyed medical students indicated that they agreed with the statement that they noticed symptoms of diseases discussed in clinical classes. Twenty-three percent of the surveyed medical students say that they do not agree with the statement. Interestingly, as many as 20% of respondents did not consider this issue.

### 3.6. Hiding Health Problems and the Field of Study

**Figure 3:**
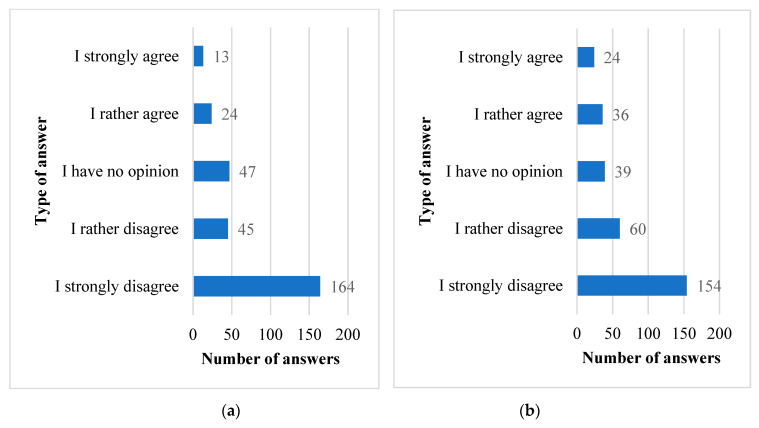
Number of responses of the surveyed students of non-medical (**a**) and medical (**b**) faculties to question the need to hide their problems due to their field of study. (**a**) Number of observations *n* = 293. (**b**) Number of observations *n* = 313.

Our questionnaire also explored that students wish to hide their health problems both amongst medical and non-medical students, due to fear of it having a negative effect in their particular field of study as shown in Figure 3. A similar number of the surveyed students of medicine and non-medical faculties (68% and 71%, respectively) declare that they do not agree with the statement regarding the need to hide their health problems due to the field of study they have taken. However, it can be noted that among medical students (20%) compared to non-medical students (12%), more people declare that they agree with the statement that they should hide their health problems due to the field of study they are undertaking.

### 3.7. What Are We Most Afraid of?

The subjects were asked to answer what diseases they are afraid of developing. The answers are presented in Table 9. It is worth noting that people who did not suffer from nosophobia and hypochondria symptoms also answered this question. Each respondent had the opportunity to provide any number of answers to this question. They were also allowed not to answer that question at all if they did not fear any kind of disease. 

## 4. Discussion

To the best of our knowledge, this study is one of the first to assess the occurrence of excessive fear related to one’s own health and the morbid belief about the occurrence of ailments among Polish students. The behavior of students of medicine at the Medical University of Silesia in Katowice in comparison to non-medical students of the University of Silesia in Katowice, the Silesian University of Technology and the University of Economics in Katowice was analyzed. The results of this study revealed that the group of medical students and the group of non-medical students achieved similar results on the nosophobic scale. These findings challenge the widespread opinion that medical students are more overly concerned about their own health than others, and debunks the common myth of the medical students’ disease by showing that it was built on erroneous beliefs. These are important findings, as persistence erroneous beliefs may lead to unjustified ignoring of disease symptoms complained by medical students leading to potentially risky consequences. As mentioned above, the initiation of such a myth took place in the 1960s [2,3], and was repeated over the next decades within the medical community. Strengthening this stereotype may be related both to the fact that medical students do acquire significant knowledge about diseases, which is why they name their ailments precisely and professionally, and due to their frequent contact with the medical staff. Consequently, they are able to consult the worries about their health on an ongoing basis and, at the same time, be noted by doctors as individuals overly worried about their own health. This is in contrast to non-medical students who are not within the medical community and have fewer opportunities to seek advice about their health. Given our findings and for the reasons discussed, in our opinion, “Medical students’ disease”/”Medical Students Syndrome” is an unfair term that may be wrongly used in a situation where a medical students seek helps for genuine worries about his or hers health.

Contrary to initial expectations, questions about hypochondria symptoms showed a greater tendency to its occurrence, not among medical students but among the non-medical students. Presumably, it results from insufficient knowledge about the ailments amongst students of non-medical faculties. This causes fear of non-specific disease entities about which these individuals have only heard from friends and families. Lack of professional knowledge can prompt research for serious causes of minor symptoms. It may also be due to the increasing spread of internet websites providing knowledge from questionable sources about diseases. Often the information that is available is not reliable in terms of content and accuracy and indeed it may also be difficult to understand by an individual not in the field of medicine [4]. Unfortunately, misinterpretation of articles on the internet may also contribute to an increase in the irrational beliefs of having a serious illness. It is also worth mentioning that individuals not in the field of medicine may not be aware of current effective treatment options for many ailments, thus resulting in elevated levels of hypochondria symptoms. Moreover, one may also be tempted to explain that medical students acquire greater and better knowledge about diseases and symptoms and therefore are likely to be more rational about their significance. Indeed, their sources (largely from academic sites) of internet medical knowledge are likely to be more reliable. The fear of being ill in our study is correlated with the year of the study. The higher the education stage a medical student is in, the greater the level of nosophobia he or she presents. However, it should be noted that the existing correlation was somewhat weak in strength. The simple explanation of this finding relates to the fact that during early years medical students focus on pre-clinical sciences and less on the clinical diseases which they learn about in more detail in the later years of their medical course. Not surprisingly, non-medical students did not show any correlation between the year of their studies and the level of nosophobia, thus confirming the above explanation of contrary results in medical students. Interestingly, the year of studies showed no correlation with the level of hypochondria symptoms among students of both medicine and non-medical faculties. It can therefore be concluded that despite the increase in the level of nosophobia among students studying medicine, the belief of being sick did not increase. This is probably due to the greater awareness with advancing knowledge amongst these students about the possibility of effective treatments being available for many diseases. It appears, increasing knowledge at higher educational levels, helps medical students to understand their perceived symptoms more rationally. 

This study also highlights other aspects of mental health, such as the frequency of mental health disorders among students. Worrying is to note, that the frequency of depression in the group of students is significantly above the average in Poland, where, according to the latest data, it affects 5.1–6% of the general population [33]. In the group of students of non-medical faculties, the declared frequency of its occurrence is 13%, while among students of medicine it is 6.7%. Equally worrying is the frequency of anxiety disorders which, according to statistics, affect 3.9% of the population [33]. In our study, they concern as many as 14.7% of students of medical faculties and 12.63% of students of non-medical faculties. It is easy to observe that they occur more than two times more often than in the general population. Among medical students, this fact may be due to the observation that 20% try to hide their problems. Therefore, they do not share their difficulties with their families and friends and therefore try to deal with them alone, which may worsen these disorders. Some respondents directly admitted that they did not seek a specialist’s help due to the fear of stigmatization. Nineteen percent of the entire group of students from our study do not plan to see a psychiatrist, although they have symptoms that may require specialist help. Efforts to reduce the fear of stigmatization can have a positive influence on the well-being of medical students [34]. We believe that greater attention should be paid to the problems of this group, especially since the number of suicide attempts has remained at a high level in Poland for many years [35,36]. According to Police statistics, in 2020 the number of suicide attempts in Poland was 12,013, of which 5165 resulted in death [37]. According to the same statistics, in the 19–29 age group there were as many as 2464 suicide attempts. Untreated mental health disorders not only hinder satisfactory academic progress, but also constitute a serious obstacle in developing skills related to future professions [38].

There is also a correlation of anxiety related to one’s own health among the group of students receiving psychiatric treatment. Significantly more often, students treated for other mental disorders declared in the questionnaire answers fear of developing a disease and hypochondria. This may result from the coexistence of certain disorders [39]. An example is the comorbidity of depression and anxiety disorders, as it is known that after an episode of depression, the probability of developing anxiety disorders throughout life is estimated at 47–58% [40,41]. Conversely, 56% of people with anxiety disorders will develop depression [42]. According to the data presented in Table 7, the greatest number of students reported a diagnosis of depression and anxiety disorders of various types, which clearly increases their susceptibility to developing nosophobia and hypochondria symptoms. This is notable, because the strength of the effect for the correlation between psychiatric treatment and the severity of fear of illness for medical students was moderate. Hence, this should be taken into account and such individuals should be offered special care. There are some possible implications of the results of our study on health policy towards medical students. The high frequency of mental health problems among them may be associated with significant distress in a medical school, as well as in other academic departments. This is why some authors analyzing this problem postulate that mental health services should be available, accessible, and affordable at universities for everyone, who needs support [43]. 

Other publications report that the female gender is relatively more often associated with a diagnosis of anxiety disorders [44,45]. Among the group of 606 students of the Silesian region, amongst the group of women, morbid fear for their own health is more frequently observed. Indeed, they are more often convinced that they have a particular disease. However, in order to unequivocally state this dependence on gender, one should carefully approach the diagnosis of anxiety disorder in this group and examine it in terms of a family burden of somatic diseases and the previous health history. It should also be remembered that women show a greater focus on their own bodies and any ailments. Indeed, they tend to respond faster to the first signs of disease compared to men. At the same time, women are characterized by a greater need to talk about their health with others. They also more often decide to contact a specialist doctor, who can address their health worries. The stereotype of men presenting to health care professionals late and less often in our culture appears to hold true [46]. It also begs the question: do women actually show anxiety symptoms related to their health more often, or do they simply admit their occurrence more frequently than men? The apparent hypochondria symptoms in women may also result from the common ignorance of disease symptoms amongst men, as the female sensitivity to disturbing signals seems to be greater.

Our study has several limitations. Firstly, it was focused on selecting students from each year of study on a one-off basis and we are aware that further research is needed to investigate the changes in the phenomenon we analyzed during the course of the university education and after graduation. Secondly, we did not use validated questionnaires in our study. As the existing validated tools did not take into account the COVID-19 pandemic, we decided to design our own tool. As we expected timely responses reflecting the current pandemic situation, the questionnaire did not go through the standard validation process. However, our questionnaire was based on DSM-5 criteria as well as on the analysis of existing literature. The way of delivering the questionnaires was also a limitation—only Facebook users could fill them in, but in the era of COVID-19, it seemed to be the safest method of collecting responses. On the other hand, according to the data from 2019 published by IAB Polska [47], there are 16,070,000 users of Facebook in Poland, the majority of whom are aged 18–34, which is the age range that covers the group of students who participated in our study. There are also available examples from the literature supporting Facebook as an effective recruitment tool [48]. Another limitation was the uneven gender distribution. It is said that female participants were more likely to fill in the forms that were sent out, and finally, the female population constituted 70% of the group of medical students. In general, according to Statistics Poland, in our country, there is a numerical predominance of female students (almost 60%) [49], and more specifically 75.6% of students at medical universities are women [50]. Additionally, according to the information received from the Dean’s Office of the Faculty of Medical Sciences in Katowice, women are 63% of students studying medicine at the University of Silesia. In Section 3.3, we made a specific gender breakdown in the parameter assessment. The analyzed samples are also not balanced by year of study, financial situation and health care type, which may have a confounding effect on our results. One of the possible reasons for the differences between the findings of our study and the results of earlier reports is the fact that students nowadays and indeed, in the last few decades, have had different access to general health education given the significant presence of the internet. Furthermore, some different personality traits can also be observed between generations of students [51]. We must also be aware of other factors, which could have an influence on the results, for example, many students of medicine are often children of physicians [52].

For our future research, we would like to expand our study population by inviting students from other universities in different regions of Poland as well as university students from another country such as the UK. This will not only afford greater statistical power but also help us to understand regional and international differences. Further understandings through future studies will be obtained by a deeper exploration of the differences in the level of nosophobia and hypochondria symptoms between men and women in both groups, as well as look for factors other than gender, which may have an impact on our observations. Stronger matching selection strategies would also be sought by taking account of factors such as greater gender balance, financial situation and access to health care.

There are some possible implications of the results of our study on health policy towards medical and non-medical students. As discussed above, the frequency of mental health problems among them is high. Therefore, mental health support should be provided for students of both medical and other faculties. As discussed above, the services offering this type of help need to be available, accessible, and affordable at different universities for all persons, who need support. Additionally introducing educational programs is required in order to increase awareness among students about the risk of incidence of mental health disorders among them as well as about the phenomenon of excessive fear related to one’s own health.

## 5. Conclusions

The findings of our study challenge the widespread belief that medical students, compared to their peers, are overly anxious about their own health. Among students from non-medical universities in Katowice, the percentage of individuals showing susceptibility to hypochondria and nosophobia symptoms is high compared to the students of medicine. Women and students receiving treatment for other psychiatric diseases are characterized by a greater fear for their own health. The prevalence of depression and anxiety disorders in the population of Silesian, Polish students seems to be significant and, therefore, there is a greater need for further research in this area. 

## Figures and Tables

**Table 1:** Cross-Section of the Group.

		Medical Students	Non-Medical Students
Sex	Female	222	223
	Male	91	70
Age	Average	21.8	21.5
	Median	22	21
Year of studies	I	84	124
	II	38	45
	III	41	41
	IV	86	49
	V	24	30
	VI	40	4
Financial situation	Very good	120	49
	Quite good	154	155
	Average	35	82
	Bad	4	7
Healthcare type	Private	79	47
	Public	71	120
	both to a comparable degree	163	126

**Table 2:** Summary of descriptive statistics of hypochondria and nosophobia variables as well as pandemic fear in the study group.

Variable under Test		*Min*	*Max*	*M*	*SD*	*SKE*	*K*	*d*
Hypochondria symptoms		7	33	12.95	4.42	0.97	0.87	0.13 *
Nosophobia symptoms		6	28	12.41	4.45	0.93	0.46	0.14 *
Fear of pandemic	Before COVID-19 pandemic	1	5	2.04	0.79	0.79	−0.18	0.67 *
	During COVID-19 pandemic	0.75	4	1.01	0.29	0.29	−0.56	0.10 *

Analyzes carried out for the number of observations *n* = 606 *Min*—minimum, *Max*—maximum, *M*—average, *SD*—standard deviation, *SKE*—skewness, *K*—kurtosis, *d*—the value of the Kolmogorov–Smirnov test * *p* < 0.05.

**Table 3:** Analysis of differences using the Student’s T test for independent data on the level of hypochondria and nosophobia symptoms between students of medical and non-medical fields.

Variable under Test	Students				*t*	*df*	*p*	*d*
	Non-Medical *N* = 293		Medical *N* = 313					
	*M*	*SD*	*M*	*SD*				
Hypochondria symptoms	13.38	4.38	12.55	4.43	**2.30**	**604**	**0.022**	**0.19**
Nosophobia symptoms	12.28	4.58	12.52	4.33	−0.67	604	0.501	0.05

*N*—number of observations, *M*—average, *SD*—standard deviation, *t*—value of Student’s test, *df*—degrees of freedom, *p*—significance level, *d*—effect size, bold highlights the most significant differences in statistical values.

**Table 4:** Summary of the correlation with the Spearman’s rho coefficient of the year of study and the level of hypochondria and nosophobia symptoms in the whole group and by fields of study.

Students	Variable under Test	Year of Study	
		*r_s_*	*p*
Non-medical *N* = 293	Hypochondria symptoms	−0.11	0.071
	Nosophobia symptoms	−0.06	0.304
Medical *N* = 313	Hypochondria symptoms	0.07	0.238
	Nosophobia symptoms	**0.13**	**0.026**
The whole group *N* = 606	Hypochondria symptoms	−0.03	0.478
	Nosophobia symptoms	0.04	0.293

*N*—number of observations, *r_s_*—Spearman’s correlation value, *p*—significance level, bold highlights the most significant differences in statistical values.

**Table 5:** Summary of the correlation with the Mann–Whitney U test of the level of symptoms and nosophobia between the studied women and men in the whole group and in the division into the research and control group.

Students	Variable under Test	Sex						*U*	*p*	*r_g_*
		Male			Female					
		*N*	*M_rang_*	*Me*	*N*	*M_rang_*	*Me*			
Non-medical	Hypochondria symptoms	70	123.77	11	223	154.29	13	**6179.00**	**0.009**	**0.21**
	Nosophobia symptoms	70	122.24	10	223	154.77	11	**6071.50**	**0.005**	**0.22**
Medical	Hypochondria symptoms	91	156.96	12	222	157.02	12	10,097.00	0.996	0.00
	Nosophobia symptoms	91	139.68	11	222	164.10	12	**8525.00**	**0.030**	**0.16**
The whole group	Hypochondria symptoms	161	282.59	11	445	311.06	12	32,456.50	0.077	0.09
	Nosophobia symptoms	161	262.41	11	445	318.37	12	**29,207.00**	**0.001**	**0.18**

*N*—number of observations, *M_rang_*—average rank, *Me*—median, *U*—Mann–Whitney U test value, *p*—significance level, *r_g_*—effect size, bold highlights the most significant differences in statistical values.

**Table 6:** Quantitative analysis of questions about admitted mental disorders.

Mental Disorders	Non-Medical Students	Medical Students
Depression	38	21
Anxiety disorders	24	20
Anxiety-depressive disorders	3	10
Neurosis	8	7
*Obsessive–compulsive disorder*	2	6
Personality disorder	7	4
Eating disorders	4	5
Panic attacks	0	3
Other	12	9

**Table 7:** Summary of the correlation with the Mann–Whitney U test of the level of symptoms and nosophobia between the subjects receiving psychiatric treatment and those not receiving treatment in the whole group as well as by field of study.

Students	Variable under Test	Psychiatric Treatment						*U*	*p*	*r_g_*
		No			Yes					
		*N*	*M_rang_*	*Me*	*N*	*M_rang_*	*Me*			
Non-medical	Hypochondria symptoms	235	140.74	12	58	172.35	14	**5344.50**	**0.011**	**0.22**
	Nosophobia symptoms	235	141.42	11	58	169.60	12.5	**5504.00**	**0.023**	**0.19**
Medical	Hypochondria symptoms	267	150.64	11	46	193.90	13	**4443.50**	**0.003**	**0.28**
	Nosophobia symptoms	267	148.20	11	46	208.09	14.5	**3791.00**	**<0.001**	**0.38**
The whole group	Hypochondria symptoms	502	290.37	12	104	366.89	14	**19,511.00**	**<0.001**	**0.25**
	Nosophobia symptoms	502	289.44	11	104	371.37	13.5	**19,045.50**	**<0.001**	**0.27**

*N*—number of observations, *M_rang_*—average rank, *Me*—median, *U*—Mann–Whitney U test value, *p*—significance level, *r_g_*—effect size, bold highlights the most significant differences in statistical values.

**Table 8:** Analysis of the frequency of responses to psychiatric treatment in relation to the perceived fear before and during the COVID-19 pandemic.

Students	Declaration on Undertaking Psychiatric Treatment				*χ* ^2^	*df*	*p*
	Before Pandemic		During Pandemic				
	*N*	%	*N*	%			
Non-medical *N* = 293	19	6.48	8	2.73	**4.48**	**1**	**0.034**
Medical *N* = 313	10	3.19	6	1.91	1.00	1	0.317

*N*—number of observations, *χ*^2^—chi-squared statistic, *df*—degrees of freedom, *p*—significance level, bold highlights the most significant differences in statistical values.

**Table 9:** Quantitative analysis of questions about concerns about the disease in question.

What Kind of the Disease do You Fear the Most?	Non-Medical Students	Medical Students
Tumor/Cancer	217	235
Psychiatric disorders	116	142
Diseases of the musculoskeletal system, e.g., degeneration of the spine	106	81
HIV/HCV/HBV infection	58	78
Cardiovascular disease	47	56
Complications of the disease I already have	39	46
Diabetes	54	46
Tick-borne diseases	45	39
Thyroid disease	39	23
Hashimoto	39	23
Neurological diseases, CNS diseases	1	9
Anemia	19	7
COVID-19	3	5
Eye-related conditions	0	5
Dermatological conditions	0	4
Parasites	0	2
Digestive system conditions	1	2
Schizophrenia	0	2
Multiple sclerosis	1	1
Respiratory system diseases	2	1
ADHD	0	1
Cold	3	0
Other advisable	diseases of the joints, sinuses, autoimmune diseases	stroke, pregnancy, alcohol dementia, motor disability, conditions that affect fertility

## Data Availability

The data presented in this study are available on request from the corresponding author.

## Supplementary Materials

The following are available online at https://www.mdpi.com/article/10.3390/ijerph18189884/s1, File S1: Questionnaire.
