# Supplementary material for: “Medical Student Syndrome”—A Myth or a Real Disease Entity? Cross-Sectional Study of Medical Students of the Medical University of Silesia in Katowice, Poland
Source: Int J Environ Res Public Health. 2021 Sep 19;18(18):9884. doi: 10.3390/ijerph18189884 (PMC8471263; doi:10.3390/ijerph18189884)
Supplement: Supplementary file 1 [file ijerph-18-09884-s001.zip › ijerph-1344334-SI.pdf]

Attachment 1

Hypochondria and nosophobia among medical students of the Medical University of Silesia

• Age

• Gender

• Year of study

• You assess your current financial situation as: very good / fairly good / average / bad

• What type of healthcare do you usually use? Healthcare private / under NHF / from both systems to a comparable degree

For questions with a scale from 1 to 5, the answers are as follows:

1-I strongly disagree
2-I rather disagree
3-I have no opinion
4-I rather agree
5-I strongly agree

1. I am afraid of getting sick. (1-5)

2. What is this fear most about?

3. Is your concern about COVID-19? Yes/No

4. Before the COVID-19 pandemic, I was afraid of the microorganisms in my surroundings. (1-5)

5. During the COVID-19 pandemic, I feel afraid of the microorganisms in my environment. (1-5)

6. Before the COVID-19 pandemic, I washed my hands excessively for fear of microorganisms. (1-5)

7. Currently, I wash my hands excessively for fear of microbes on them. (1-5)

8. I often notice symptoms of a given disease after completing classes on them. (1-5)

9. After the clinical course, I start to pay more attention to the symptoms of diseases related to the topics of the classes/patients I studied. (1-5)

10. Suspecting disturbing symptoms causes me fear. (1-5)

11. It happens that I underestimate the disturbing disease symptoms. (1-5)

12. Fear of illness keeps me awake/wakes me up at night. (1-5)

13. I have panic attacks (palpitations, chest pain, shortness of breath, nausea) related to

fear for their own health (1-5)

14. If you selected 2-5 in the previous question, which symptoms accompanies you when do you think about the disease for which you feel the most fear? Dyspnoea / chest pain / heart palpitations / rapid breathing / shortness of breath / derealization / depersonalization

15. In which year of your studies did you start noticing the above symptoms? (only in the case of marked the answer in the question above)

16. Fear of illness makes it difficult for me to function in socjety. (1-5)

17. I am convinced that I have some serious illness. (1-5)

18. I sometimes think of symptoms that indicate a medical condition that I suspect in myself. (1-5)

19. I usually take steps to diagnose the disease that I found out about and which I have had I suspect. (1-5)

20. I often visit a doctor with a suspicion of a disease that I myself have diagnosed with. (1-5)

21. I always trust my doctor when he denies that I have any disease which I suspect. (1-5)

22. I often look for the opinion of many specialists if the previous ones denied my disease which I suspect at home. (1-5)

23. I often confide in friends and acquaintances with concerns about my health. (1-5)

24. I believe that I should hide my problem because high demands are placed on me due to the studies I have undertaken. (1-5)

25. During the Sars-CoV-2 pandemic, the symptoms of the disease I suspected worsened. (1-5)

26. Before the COVID-19 pandemic-did you use therapy due to the fear of getting sick? Yes/No

27. During the COVID-19 pandemic, did you use or are currently receiving therapy in relation to the feeling of fear of getting sick? Yes/No

28. Are you planning to visit a psychiatrist/psychologist because of your fear of getting sick?

Yes / no, although I do feel anxious / no, I do not feel anxious / maybe

29. If in the question 28 you marked the answer: "no, although I do feel anxious": Why are you not planning to visit a psychiatrist /psychologist in connection with the perceived fear of getting sick?

The visit exceeds mine financial possibilities/I am afraid of stigmatization/I can handle myself/another answer

30. Do you use psychiatric /psychotherapeutic counseling for other reasons? Yes/No

31. What other mental disorders do you suffer from?

Hypochondria and nosophobia among students of non-medical faculties

• Age

• Gender

• Are you a student of the Silesian University of Technology?

• Are you a student of the University of Economics in Katowice?

• Are you a student of the University of Silesia in Katowice?

• Year of study

• Field of study

• You assess your current financial situation as: very good/fairly good/average/bad

• What type of healthcare do you usually use? Healthcare private/under NHF/from both systems to a comparable degree

• Are your parents working in a medical profession? Yes/No

For questions with a scale from 1 to 5, the answers are as follows:

1-I strongly disagree
2-I rather disagree
3-I have no opinion
4-I rather agree
5-I strongly agree

1. I am afraid of getting sick (1-5)

2. What is this fear most about?

3. Is your concern about COVID-19? Yes/No

4. Before the COVID-19 pandemic, I was afraid of the microorganisms in my surroundings. (1-5)

5. During the COVID-19 pandemic, I feel afraid of the microorganisms in my environment. (1-5)

6. Before the COVID-19 pandemic, I washed my hands excessively for fear of microorganisms. (1-5)

7. Currently, I wash my hands excessively for fear of microbes on them. (1-5)

8. I often notice symptoms of a given disease after reading about them on the internet or in the newspaper. (1-5)

9. Suspecting disturbing symptoms makes me fear. (1-5)

10. I sometimes downplay disturbing disease symptoms. (1-5)

11. Fear of illness keeps me awake/wakes me up at night. (1-5)

12. I have panic attacks (palpitations, chest pain, shortness of breath, nausea) related to fear for their own health. (1-5)

13. If you selected 2-5 in the previous question, which symptom accompanies you when do you think about the disease for which you feel the most fear? Dyspnoea/chest pain/heart palpitations/rapid breathing/breathlessness/derealization/depersonalization etc.

14. During which year of your studies did you start noticing the above symptoms? (only in the case of marked the answer in the question above)

15. Fear of illness makes it difficult for me to function in socjety. (1-5)

16. I am convinced that I have some serious illness. (1-5)

17. I sometimes think of symptoms that indicate a medical condition that I suspect in myself. (1-5)

18. I usually take steps to diagnose the disease that I found out about and which I was having I suspect (1-5)

19. I often visit a doctor with a suspicion of a disease that I myself have diagnosed with. (1-5)

20. I always trust my doctor when he denies that I have any disease which I suspect (1-5)

21. I often seek the opinion of many specialists if the previous ones denied that I have a disease which I suspect at home. (1-5)

22. I often confide in friends and acquaintances with concerns about my health. (1-5)

23. I believe that I should hide my problem because high demands are placed on me due to the studies I have undertaken. (1-5)

24. During the Sars-CoV-2 pandemic, the symptoms of the disease I suspected worsened 1-5

25. Before the COVID-19 pandemic-did you use any therapy due to the fear of getting sick? Yes/No

26. During the COVID-19 pandemic, did you use or are currently receiving therapy in connection with the feeling of fear of getting sick? Yes/No

27. Are you planning to visit a psychiatrist /psychologist because of your fear of getting Sick? Yes/no, although I do feel anxious /no, I do not feel anxious / maybe

28. If in question 27 you marked the answer: "no, although I do feel anxious": Why are you not planning to visit a psychiatrist /psychologist in connection with the perceived fear of getting sick?

The visit exceeds mine financial possibilities/I am afraid of stigmatization/I can handle myself/another answer

29. Do you use psychiatric/psychotherapeutic counseling for other reasons? Yes/No

30. What other mental disorders do you suffer from?
